# Communication between parents and neonatal healthcare professionals using pictorial support when language barriers exist – parents’ experiences

**DOI:** 10.1080/17482631.2022.2122151

**Published:** 2022-09-07

**Authors:** Eva-Karin Gotting, Ulrika Ferm, Helena Wigert

**Affiliations:** a Institute of Health and Care Sciences, Sahlgrenska Academy, University of Gothenburg, Gothenburg, Sweden; b Division of Paediatrics, ANS Hospital, Angered, Sweden; c DART Centre for Augmentative and Alternative Communication and Assistive Technology, Sahlgrenska University Hospital, Gothenburg, Sweden; d Division of Neonatology, Sahlgrenska University Hospital, Gothenburg, Sweden

**Keywords:** Communication, interpreters, interviews, language barriers, pictorial support

## Abstract

**Purpose:**

Families arriving in Sweden after being forced to flee their home need health care. Communication is a key component to establishing good care relations and becomes difficult when there are language barriers between families and healthcare professionals. In the context of neonatal care, communication is carried out with parents. The aim of the study was to describe parents’ experiences of communication with neonatal healthcare professionals and using pictorial support when language barriers exist.

**Method:**

The study takes a qualitative approach based on seventeen interviews with parents who had experienced neonatal ward. Qualitative content analysis was used.

**Results:**

The parents needed to communicate through supports, which caused distress and misunderstanding. The relationship between parents and the healthcare professionals affected the communication. Pictorial support was used to different degrees. Four categories were identified from the data analysis: Communicating through supports, Facing barriers in communication, Facing external influences and The need for a good healthcare relationship.

**Conclusion:**

The present study is the first to describe parents’ experience with using the pictorial support developed in the project KomHIT Refugee and therefore fulfils the function of being a first evaluation of the pictures from parents’ perspective.

## Introduction

An important part of neonatal care is the relationship between the paediatric nurse and the newborn child’s family. Maintaining children’s and parents’ trust might be challenged by the fact that the nurse often must conduct procedures that are unpleasant for the children. In these kinds of situations, it is important that the children and parents are as involved as possible so that they feel prepared for the procedure (Coyne et al., 2016). The Swedish Health Care Act (2017) highlights the importance of healthcare providers taking parents into account when a child is ill. Parents have the right to be part of the healthcare configuration when their child is hospitalized (a.a).

Establishing good care relationships between families and healthcare professionals can be difficult when language barriers exist. Communication is an important component within the care relationship, and if it does not function well, there is a risk that the families may feel that the health care provided is not satisfactory (Kroening et al., 2016). Language barriers is found to be the most common influencing factor for communication problems (Kaufmann et al., 2020). Earlier studies describe that staff within neonatal care experience frustration and powerlessness when they communicate with parents where language barriers exist. They found their own strategies to communicate, using body language and assistive technology (Patriksson et al., 2017). A field study shows that health care professionals preferred to use an interpreter when language barriers exist, while parents in neonatal care wished to speak for themselves or asked for multilingual professionals to interpret (Patriksson et al., 2019).

Simplified language and pictorial support are studied as potential ways to communicate more effectively and thereby secure the quality of health care for patients with both high and low health literacy (Meppelink et al., 2015). Projects where pictorial support is used to facilitate communication with refugees in different contexts are ongoing around the world (ICOON for refugees, 2021). KomHIT Refugee (2018) is one example which has been implemented in healthcare facilities in Sweden. KomHIT Refugee seeks to facilitate information exchange and communication between healthcare professionals and patients in care situations. Based on statistics from the Migration Authority, the pictorial support is translated to eleven languages common among asylum seekers in Sweden. The pictorial support is audited by medically knowledgeable healthcare professionals. All healthcare units that have participated in the KomHIT Refugee project, such as the Neonatal Intensive Care Unit (NICU) where this study was conducted, have identified care situations where they find there is a need for pictorial support. According to Swedish law, anyone in need of an interpreter is entitled to one when in contact with authorities (SFS, 2017). The pictures are used as a complementary support when communicating with an interpreter (a.a.).

Language barriers challenge family-centred care and research is needed exploring parents’ perceptions regarding care by other-language spoken health professionals (Stephen, 2021). There is a lack of knowledge regarding parents’ experiences from communicating with neonatal health professionals and using pictorial support when language barriers exist.

## Aim

The study had two aims: to describe parents’ experiences of communication with neonatal healthcare professionals when language barriers exist, and to describe their experiences of using the KomHIT Flykting pictorial support.

## Method

A qualitative content analysis according to Graneheim and Lundman (2004) was the design of the study, and the results were construed from narrative interviews conducted with semi-structured guidelines.

## Settings

The study commenced with a literature review focusing on newly arrived patients’ needs in Swedish health care, as well as earlier studies about pictorial support within health care. Six months before the first interviews took place, the first author (E-KG), together with a project worker from KomHIT Refugee, visited two workplace meetings on neonatal wards in a university hospital from which the study participants would be recruited. The purpose of these visits was to inform the healthcare providers in these wards about the research subject and engage the medical staff to use the pictorial support in care interactions as much as possible. These meetings were intended to increase the chances for prospective participants to be exposed to pictorial support before upcoming interview sessions where the author would ask questions about the participants’ experiences with the pictorial support. The author then kept in contact with one person in each neonatal ward to regularly remind the medical staff about using the pictorial support and about the ongoing research project.

## Ethics

This study received approval from the Ethical Review Board of Gothenburg (ref:102–18) and obtained consent from the operations manager of the Care Unit. The participants were informed that personal data would be anonymized and that they could withdraw their data at any time during the research process without having to give any special reason. All participants in the study gave their written consent to participate in the study.

## Participants

Seventeen mothers, twelve fathers and two other family members whose native language was not Swedish, connected to a Neonatal Intensive Care Unit (NICU) in southwestern Sweden, entered the study (Table 1). The inclusion criteria for participation were: at least one of the parents was not Swedish speaking, their child had been hospitalized for at least a week in one of the units, and that they had been discharged one to six months before the interview session.

**Table 1.** Demographic data (n = 17).

Families	Number
Interview with both parents	12
Interview with mother	3
Interview with mother and one other family member	2
Mother’s age in years, mean (min-max)	25 (19–40)
Father’s age in years, mean (min-max)	30 (19–48)
Other family members’ age in years, mean (min-max)	46 (20–52)
Years living in Sweden at time of interview, mean (min-max)	3 (0–10)
Exposed to KomHit Flykting pictorial support	
Never	15
Rarely	7
Often	7
Professional interpreter participated in the room	9
Professional interpreter on telephone	3
Parents preferred to speak English	2
Parents wanted partner/friend to interpret	3
Length of interviews	
Less than 30 minutes	5
30–44 minutes	8
45–60 minutes	2
More than 1 hour	2
Languages spoken by parents	
Albanian	2
Arabic	4
Gujarati/English	1
Kurdish	2
Malyalam/English	1
Somali	11
Sorani	3
Urdu	2
Vietnamese	2
Tagalog/English	1

## Data collection

When the operations manager approved the study, the NICU’s secretary gave the first author contact details of caregivers. Parents were then contacted by telephone by professional interpreters who were not part of the research team and informed verbally about the aim of the study. If they agreed to participate, the author called back, with an interpreter, to provide further information about the study and to book a time and place for the interview with the approval of the caregivers. Before the interview started, the parents signed a consent form indicating their agreement to participate. The interviews were held in private locations, to meet the confidentiality needs of the parents.

The author is a specialist in paediatric nursing and has experiencefrom communicating with parents within health care.

In most interviews, both parents were present, although in some cases there was one parent or another family member who had also been present during the hospitalization at the NICU. Most caregivers agreed to having a professional interpreter at the interview sessions. Some preferred one of the partners to interpret to the other, or else they wanted a friend to interpret for them. Most interviews took place in the homes of the families. One parent wanted to meet at a public library. The length of the interviews was 19–72 minutes (Table 1). The interviews were digitally recorded and transcribed verbatim.

A semi-structured interview guide was used with open questions to create a conversation where the parents could feel free to share their experiences. The author encouraged the parents to speak freely and with their own words regarding the research subject.

## Data analysis

The interviews were analysed according to qualitative content analysis, with an inductive approach (Graneheim & Lundman, 2004). The interview transcripts were read though several times by the author to obtain a general sense of their content. Manifest content was studied, based on what was concretely said in the text. The text was studied in its entirety and then broken down into smaller units. The meanings of the units were condensed into smaller units of text that still contained the essence of the data in its original form. Every condensed unit was labelled with a code name. The codes created categories with different subcategories based on related content (Table 2). In total, four categories, each with two to three subcategories, emerged from the data analysis.

**Table 2.** Examples of analysis process.

Unit of meaning	Condensed unit of meaning	Code	Subcategory	Category
What they are saying of course they are doing for the good of my daughter so I should be taking care of it, whatever they are saying exactly, because they want us to be better and go from here, I understand that.	they are doing for the good of my daughter, I understand that.	Sense that staff is doing good.	Feeling trust and confidence.	The need for a good healthcare relationship.
Sometimes, someone who talks to me really quick and I say “Yes, I understand,” but from inside I don’t understand, I don’t understand anything and I get really mad at myself because of this, that I don’t understand. It’s really, really tough actually.	I say “I understand” even though I don’t and I get really mad at myself and it’s really tough.	Feeling it’s tough and feeling mad at oneself.	Facingmisunderstandings.	Facing barriers incommunication.

The categories included all data in the text that responded to the aim of the study, and no parts of the text were excluded due to the lack of a suitable category. All data concerning the parents’ experience of communication with healthcare providers when language barriers existed at the NICU were included in the coding as being potentially relevant. No data were placed into two different categories; all relevant data were placed into a suitable category.

## Results

**Figure 1.**
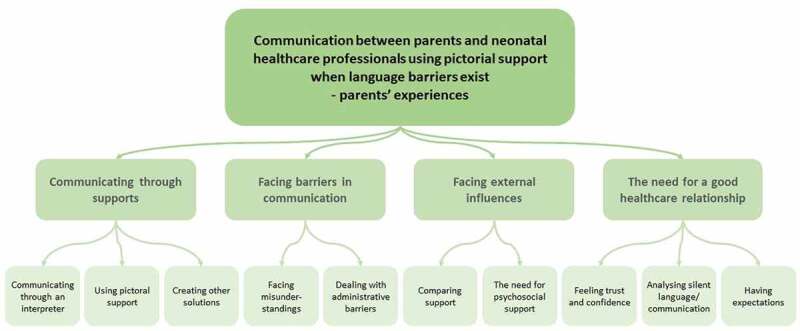
Overview of the findings, categories and subcategories.

Analysis resulted in four categories describing parents’ experiences of communication with neonatal healthcare professionals and using pictorial support when language barriers exist. Each category has two to three subcategories (Figure 1).

## Communicating through supports

Parents in the study discussed different types of supports regarding communication that they had been introduced to during their child’s hospitalization at the NICU.

### Communicating through an interpreter

All parents mentioned that they had communicated with healthcare professionals through an interpreter at some point during their child’s hospitalization. Whether or not an interpreter was present could depend on which category of healthcare professionals the parents were meeting with. Interpreters were not booked frequently for when nurses were communicating compared to when doctors were. One reason mentioned to this was that the nurses talked rather slowly and explained things in a careful way. Parents felt that when they were talking to the healthcare professionals working daily at the NICU, the nurses were listening carefully to what the parents said and that the nurses tried to understand them. Another reason was that parents sensed that the subjects they were talking about with the nurses were rather simple, concerning milk and diapers, for example, and for these no interpreter was needed. They thought it was not necessary to always have an interpreter, but it was when it concerned special meetings and important information. Some parents also mentioned that they did not want any interpreters booked because of the cost; they thought that the money spent on interpreters could have been saved for other purposes in the NICU. There were situations described by parents where, in a care meeting, the interpreter did not speak the same dialect as themselves. Sometimes, too, the interpreter did not understand the dialect of the doctors they were interpreting for. This would sometimes lead to severe misunderstandings that made the parents feel overwhelmed.

*“The interpreter that you book, you have to be sure that they have the right education and are able to interpret the information very well. If the interpreter, for example, says that you have a serious disease, it can be the baby or yourself. But maybe that’s not right, maybe the interpreter interpreted wrong. When you are shocked, you can be deaf to what is being said.”* (Parent 1)

In some cases, it helped if someone else in the room besides the interpreter was explaining what the healthcare professional said. Sometimes, after a disappointing experience with one interpreter, parents found that a different interpreter present at their next meeting worked out fine, and they were relieved to know that that interpreter would be booked at their meetings thereafter.

Other parents described that a healthcare professional acted as the interpreter initially at the hospital stay. Most commonly, it took a few days before a professional interpreter was booked. Parents thought that with the professional interpreter, they were able to communicate, explain more, and ask more questions and that there was more time for this communication. However, parents expressed that there was greater understanding regarding medical vocabulary when a healthcare professional, with the same mother tongue as the parent, was interpreting. They also revealed that it took longer to communicate through an interpreter. Sometimes the interpreter was booked to communicate by telephone.

*“It might be difficult sometimes, because it takes longer. Sometimes it is also difficult to hear through the telephone and you often have to wait for the interpreter.”* (Parent 8)

### Using pictorial support

Some parents had not experienced the use of pictorial support such as KomHIT Refugee, but they described experiences from care meetings where they would have liked to use pictorial support and thought positively about this communication tool. Parents thought pictorial support would be helpful, for example, when providing information about the kinds of items that would be needed for the hospital stay, such as blankets or pillows. Parents explained that not being able to ask for certain things caused feelings of loneliness.

*“Sometimes I felt alone. When I couldn’t explain something and when I didn’t have a picture to show for explanation. At the hospital there is a bureau with clothes and diapers and stuff, and it was labelled in Swedish but there were no pictures if you couldn’t understand Swedish.”* (Parent 5)

Parents who were introduced to pictorial support explained that the interpreter would go over the pictures and that the pictorial support was in their room during the hospital stay.

*“We got the pictorial support from the hospital to help with communication. To communicate, they fetched one of these and they said, “What you want, you can point to. They even had it in Arabic so that I could point to the picture. I had a schedule with pictorial support in my room at all times until we were discharged. Without the pictures, I would need an interpreter at all times, you could say.”* (Parent 2)

It was revealed that some parents learned Swedish words from the pictorial support. Other participants mentioned that the pictorial support was helpful, but not enough. They requested larger and more pictures with further explanations, and the participants said that they found the pictures to be most helpful when communicating about simpler subjects.

*The pictures helped me a lot when I needed supplies in our room or when my husband was not there and I was in need of the bathroom. When the doctor came, he asked deeper questions than the pictures could be used to answer, and the doctor was not often there … ”* (Parent 17)

Some parents suggested that there should be pictures in the reception area and on different bulletin boards in the wards to aid with communication and understanding. Other parents also suggested that the pictures should always be labelled in English as well, since the labels are not translated into all languages.

It was also revealed that some parents had been exposed to pictorial support in other contexts before their hospitalization but did not see the pictures at the NICU. Other parents explained that when they were discharged from the hospital, they got the information together with pictures describing how to proceed at home with their child.

*“We got them before his surgery with information on how the procedure would be done. And then when we left from surgery after one day, we got a rather big paper with several pictures explaining what he couldn’t do, for example, that he couldn’t run or jump.”* (Parent 10)

### Creating other solutions

Sometimes neither interpreter nor pictorial support was available when communication was nevertheless needed. The parents described different types of solutions that they took into care relationships to support communication.

If one partner could speak English, parents mentioned that they used English when their Swedish was limited. At other times, parents used the internet to search on Google for translations to help them communicate with the healthcare professionals. It was not mentioned whether the healthcare professionals used this approach. Calling a friend and either asking them to translate a specific word or letting them speak to the healthcare professional directly was also mentioned in the interviews as a way of facilitating communication. Some parents mentioned that when they discovered something new with their child, they recorded it with their mobile camera so that they could show the healthcare professional the video instead of trying to explain.

Parents also mentioned that they and the healthcare professionals both used their hands to communicate by making gestures and pointing to different things. Parents described these as common ways that people communicate with each other when they do not speak the same language and that one can manage that way.

*“For example, this picture was not there* (pointing at a picture of a baby being bathed brought by the author to the interview); *‘shower the baby’; this picture was not there, and every week we had decided, but when she talks; I don’t understand, but when she says “Shower” and points to her head, then I understand.”* (Parent 2)

Some parents explained that, before visiting hospital, they had looked up words they thought they would need to know in order to understand information given there, and to make themselves understood.

## Facing barriers in communication

Different types of factors emerged that led to challenges in communication. Even though parents were very satisfied with their time spent at the NICU, distressing events caused by communication barriers were described in the interviews.

### Facing misunderstandings

The parents described different kinds of misunderstandings that occurred during conversations, which caused unnecessary stress. The misunderstandings occurred for different reasons, such as the interpreter misinterpreting a word so that parents did not get an accurate understanding of the situation. The misunderstandings caused worries and stress about their child’s health. These misunderstandings also led to feelings of discouragement because the parents felt that they and the healthcare professionals did not understand each other well enough. One mother described how her already high blood pressure went even higher after facing misunderstandings in communication with healthcare professionals. In other cases, parents felt that the healthcare professionals misunderstood or that different staff members gave different advice.

*“In certain situations, it happens that we misunderstand and we are trying to find a proper conversation so that we can explain ourselves and why something has happened, for example, why I was half an hour late in feeding the baby, because she was sleeping so soundly. I just simply say that ‘she was sleeping so soundly’. The nurse would not understand; she was like, ‘You should wake her up, you should feed her. You know, these times are very important, every three hours she should have a feeding.’ Then I also have to explain to her that, you know, ‘she was very sleepy’. I went to the other nurse, I explained to her, and she said, ‘Wait another half an hour; she will wake up if she is hungry.”* (Parent 6)

When talking about communication, emotions were expressed. One mother explained that when a person is shocked, they can become deaf to anything said right afterwards. Other feelings included a fear of being misunderstood.

*“I got very scared. How am I supposed to tell them that my daughter has pulled out her feeding tube?* (Parent 7)

### Dealing with administrative barriers

Parents revealed that not having an internet connection affected communication with healthcare providers. Other barriers to communication included a lack of pictures on signs at the hospital, and a lack of written instructions in languages other than Swedish.

*“’Cause when I buy the medicine from an international market, I found that there are many languages; the whole-country language is there, but English is always, always, always there. So many times, it happens here that we do not have that in the instructions. For that particular medicine, I have to search in Google. Many of them* (other immigrants) *are not so educated, they just blindly follow what they understood, and sometimes it can be harmful for the child. So, I think it’s very important to add more languages in the product information.”* (Parent 6)

## Facing external influences

Even though the author of the study did not ask questions regarding the parents’ conditions outside their child’s hospitalization at the NICU, this was brought up by the parents themselves in relation to communication. They also discussed what they had heard friends and relatives talking about concerning communication in other situations and how they perceived that supportive communication is being discussed in other places outside the NICU.

### Comparing support

When parents talked about friends’ experiences with Swedish health care, they were similar to their own experiences. Also, when talking about communicating with other authorities in Sweden, such as the Migration Authority and universities, they perceived that authorities perform their duties well and communicate in similar ways as they do within health care. There were other experiences with communication they encountered in other parts of society, and they had thoughts about how to integrate similar supports into health care.

*“I always use these kinds of apps, like Google Translate and also another app. But I mean that this should be installed on a screen. It would be just a small screen located maybe at the reception. When certain persons come in, you ask them to write.”* (Parent 6)

### The need for psychosocial support

Some parents expressed the importance of the healthcare system to understand that patients like themselves are in a unique situation. Apart from being ill, or having a sick child, with all the feelings that come with that, they also cannot speak the same language as the healthcare professionals. Another psychosocial condition that parents mentioned as affecting communication was whether or not it was their first and only child that was hospitalized. Parents found it difficult not to spend all their time at the hospital. They expressed that they had feelings of loneliness and frustration when they got home; that they felt they could do nothing at home, and they were scared of what would happen when they left the hospital. Some parents lived in the suburbs at a long distance from the hospital and revealed that they did not have a car or had not been granted asylum, which exacerbated feelings of isolation and anxiety. Some enabling factors were also mentioned; for example, that this was not their first time in a new country, they were used to coping with different situations that occurred in their new society and healthcare system.

*“We had our children after we had been living here for some years, so we were not completely blank when we came here, but we didn’t understand as much as now.”* (Parent 9)

Some parents explained that it helped a lot when staff enabled the parents to go home to their other children. They expressed the importance of the staff considering everything regarding this break from the hospitalization, helping the parents feel that it was safe to leave their sick child at the hospital for a while.

## The need for a good healthcare relationship

Throughout the interviews, parents talked about other ways to communicate besides verbally, and about feelings that affected patients’ perceptions around communication.

### Feeling trust and confidence

Parents felt that they knew the Swedish language, but when it came to situations regarding health care, they felt less confident. They felt responsible for the care of their child since they knew their child better than the healthcare providers did. When the child’s health status changed, parents felt obligated to explain these improvements or impairments. When at home and when they went to follow-up appointments at outpatient clinics, parents noticed that they could express themselves better and that the outpatient clinics chose not to book an interpreter. After a meeting with no interpreter, parents felt empowered and confident. Also, some expressed that they were happy when they were able to offer a correction when the interpreter was wrong. Another factor that had an impact on the parents’ confidence was when they learned new words from the healthcare professionals, expanding their vocabulary during their time at the NICU. Some parents also explained that the healthcare professionals informed them that the videos they had made of their child’s improvements and impairments were shown to other experts at the hospital so that they could learn from each other, which encouraged the parents and assured them that what they were doing was good. Parents also explained that they felt joy and felt encouraged by the staff when they were making eye contact and telling them that they were good parents and that their child would be better soon.

*“Every time I talked with them* (healthcare staff), *the pain was relieved. They were really kind … I was comforted by them and able to hug them; it felt like they were part of my family.”* (Parent 16)

Some parents expressed appreciation and trust when the healthcare staff noticed that they were not able to follow the conversation in Swedish and switched instantly to English; sometimes this was enough rather than talking in their mother tongue.

*“I think it’s fine because they also talk in English and understand my situation.”* (Parent 14)

*“They don’t say anything just because you can’t speak Swedish. We had so many questions and so many things that we were not sure about: how it’s gonna happen, but everything was explained to us so well and we didn’t have any doubts.”* (Parent 15)

Other experiences revealed the opposite, when no trust was established between parents and healthcare professionals, and the communication was clearly affected.

*“She had just started and knew nothing about what we had done before. That has to be communicated between the nurses before, I think. Not by us. We knew what was best for the child, but we sensed that the nurse did not believe so. I felt that I did not care about her; she only said something quickly and then disappeared again. I listened to someone else instead.”* (Parent 4)

### Analysing silent language/communication

Some parents mentioned that communication is not only about words. They felt, for example, that they could tell whether a nurse was attentive when communicating by the way the nurse was looking at them and if eye contact was established. Different types of body language were important for the parents to decide whether they liked the nurse or not, which had significance in terms of whether or not they trusted the nurse.

*“We talked like usual, like we talked with the others. But it felt as if she was standing aside and talked just a little and then left from there. In the beginning, she came with someone else, but every time when she came alone this happened; she was not comfortable somehow.”* (Parent 4)

Other parents explained that they were more likely to feel understood if the person they were communicating with acted in an attentive way, that this helped to make communication positive. They also explained that they felt happier and calmer when staff helped out with more than they were obliged to, for example, by providing a sandwich if they noticed that the parents had not eaten for a long time.

*“It is not easy to talk if you feel that someone is annoyed at you … One nurse was good … she helped me so that I could have him in my arms, even though he was getting his light treatment … To bring the lamps for me and do some things that were kind of extra, not only what was convenient for them (the staff) but also taking what I want into account. With compassion you come far when you don’t understand each other.”* (Parent 13)

Other parents indicated that some silent communication is similar in all languages.

*“There are certain things that are similar in all languages when it comes to body language and signs; for example, when they told me to wait or explained to me that it was time for breastfeeding, these kinds of things I could understand when they were instructing me.”* (Parent 12)

### Having expectations

Parents revealed that when they knew they were scheduled to have an information meeting at the NICU that included an interpreter, they did not sleep well the night before as they had expectations and enquiries before the meeting. They expressed that when an interpreter was booked, they expected that the information was going to be important. Therefore, it was possible that misunderstandings could arise that may be difficult to deal with. Parents also expressed that they felt powerless regarding how the healthcare professionals would provide the information—whether it would be given with or without an interpreter, or with or without pictorial support. Parents did not feel comfortable asking about how the information would be provided, and they did not feel that they had the right to make that kind of request. Further expectations among parents included assumptions that healthcare professionals could speak English along with more general thoughts around health care, such as that there may be delays due to staff shortages or that they might have to speak up in order to be listened to.

*“Sometimes it feels like if you don’t insist, they can be a bit ignorant or not take things seriously. What I mean is that if you can communicate and stand up for yourself, they will listen better to you, and of course the language matters in this matter too.”* (Parent 12)

Other parents revealed that they were surprised about the great health care provided to their child since their own health had not been taken care of since their arrival to Sweden because they had not yet attained legal resident status.

## Main interpretation and discussion

When parents were asked about their general experience regarding communication with healthcare professionals during their child’s hospitalization at the NICU, most indicated that they were very satisfied. Despite this, parents also revealed distressing experiences caused by misunderstandings in communication. Parents expressed that they felt calm when healthcare professionals were comforting them, and this could possibly have contributed to the overall positive experience of communicating with healthcare professionals. Similar findings were made in another Swedish study about experiences of communication with pictorial support among children aged 7 to 13 (Benjaminsson & Nilsson, 2016). Even though the study was based on Swedish-speaking children, the similarities regarding feeling a sense of encouragement, trust and confidence were striking, consistent with the adult non-Swedish-speaking parents in this study. In both studies, the participants thought that it was reassuring when healthcare professionals were saying that all would be well and that they shouldn’t worry about the medical procedures. The information that was given to participants in the study conducted by Benjaminsson and Nilsson (2016) helped the children to calm down (a.a). Parents in the current study explained situations where they had different stressful perceptions about their child’s condition. When a healthcare professional refuted these assumptions, the parents felt relieved. Thus, the barriers to communication were first experienced as parents worried about their child, but when they were informed about their child’s ongoing medical treatment, they could let go of their worries and were more amenable to communication. Parents mentioned that it was more likely to establish good communication if they felt that the healthcare staff acted in an attentive way. The results from a study in Switzerland explains that nurses felt frustrated when they were not able talk to patients but only comfort them in other ways (Kaufmann et al., 2020). Present study establish that this kind of comfort is valuable to enable trust and well-functioning communication.

In our study had not all participants been exposed to the pictorial support distributed within the project KomHIT Refugee (2018). There might be different explanations for this according to a study that aimed to investigate the early implementation process of pictorial support in neonatal care from a healthcare professional’s perspective (Blom et al., 2019). Even though the healthcare professionals were motivated to use the pictorial support and experienced a great need for it, the results revealed that education and having someone formally in charge is an important part of making the implementation successful (a.a). Considering both the context and year of the above-mentioned study, it is very likely that these reasons would be applicable to the present study as well.

The parents explained that when one or both partners had not yet obtained asylum, this would be a stressful factor that could affect communication. This might be because these participants are more likely to have lower health literacy compared to those who have obtained asylum. Limited health literacy amongst asylum seekers is described in a review article of qualitative studies; healthcare provision might be challenging when there are different understandings of health, illness and health care (Robertshaw et al., 2017). Healthcare concepts such as preventative care were also described as sometimes unfamiliar, making patients that are asylum seekers prone to missing appointments (a.a). Similar findings in the present study indicated that parents did not understand why they were sent messages to come to the hospital for follow-up treatment when they felt that their child was well and healthy. They explained that they did not understand why they had to go back to the hospital. Therefore, knowledge and understanding about the cultures of refugees and asylum seekers are viewed as important facilitators in cross-cultural care (Robertshaw et al., 2017).

Parents in the present study described stressful situations when they were told by healthcare professionals to go home for the night. They expressed that they lived far away from the hospital and felt stressed about what would happen when they were not with their child at the hospital. Other stressful factors might be anxiety about how to get back and forth between home and hospital when they did not have an identification number to give should a ticket inspector ask for identification. This stress before appointments with healthcare providers could affect communication, as explained above. Parents in the present study described feelings of relief when the healthcare professionals let them stay overnight. According to Robertshaw et al. (2017), one way to facilitate a trusting relationship between patients that are asylum seekers and healthcare professionals is for healthcare professionals to assist the patients with their wider needs, such as by enabling the parents to stay with their child at the hospital overnight if needed (a.a). This is similar to the findings of this study, where the parents described a good healthcare relationship as a factor that affects the communication with healthcare professionals when language barriers exist.

The parents did not feel that they had the right to ask whether or not the pictorial support would be used or if an interpreter would be booked. They claimed that this decision was in the hands of the healthcare professionals. This can be discussed as a consequence of low health literacy amongst participants who are not aware of their rights, but also as a failure of healthcare professionals in meeting the patients’ needs to understand their right to information. According to results from a Swedish study nurses tend not to be trained in using interpreters (Jungner et al., 2021). If healthcare professionals, specifically nurses, are to take a key role in helping migrants acquire health literacy, it is important to consider the changes to clinical best practices that would be required to strengthen both the level of health and legal literacy for migrants and also the pedagogical skills of nurses (Vissandjée et al., 2017). Some parents in the present study experienced that they felt more confident in expressing themselves when visiting health care outside hospital. This might be the result of having spent more time in the country and not having to face as stressful health care as at the NICU, and therefore having higher health literacy and being more communicatively adaptable, but it may also be a result of the nurses having more time in non-acute outpatient clinics to take a more pedagogical role (a.a).

## Methodological reflections

Some parents wanted a professional interpreter present during the interview but indicated that one of the partners always acted as the interpreter during the hospitalization at the NICU. Other parents wanted an interpreter during the interview but told the author that during hospital visits they did not use interpreters. A possible reason for this is that when it comes to deeper conversations there is greater need to speak in their mother tongue. Parents expressed feelings of frustration, even during the interviews, that they were not able to speak in their mother tongue since they felt sometimes that the interpreter was not interpreting correctly. Some parents wanted an interpreter on the phone because they didn’t want to have a lot of visitors in their home at the same time, while other parents preferred not to have an interpreter at all but wanted to carry out the interviews in English. When the interviews were in English, and no interpretation was done, the length of the interviews was rather long compared to others. This might indicate that when the author and the parents speak the same language, it is likely that both parties feel freer to speak directly to each other and greater communication is enabled. When speaking the same language, even though it is neither the author’s nor the parents’ first language, the possibility for a parent to interrupt a question and lead the conversation in another direction is greater. All parents were offered an interpreter. When it became clear that the desire to have an interpreter present differed from parent to parent, the author chose to let the parents decide for themselves. The author considered that this could have affected the study, but not necessarily in a negative way. Most importantly, the parents had to feel comfortable with how the interview was being conducted, otherwise, the absence of comfort would likely have greater negative effects on the study.

Difficulties with qualitative content analysis include different ways of interpreting the data, and the probability that the interpretation will be influenced by who is reading the text and analysing the data (Graneheim & Lundman, 2004). There are limitations in this study since there were fewer possibilities to collect additional data due to language barriers. For the same reason, it was difficult for the author to establish member checks, where the parents review the interviews and the analysed data. Member checking is done to fulfil credibility as one of the components to achieve trustworthiness in qualitative studies, as described by Guba and Lincoln (Polit & Beck, 2016). Thus, the author strove for credibility through triangulation by asking the same questions to all parents in the study. A semi-structured interview guide was used to keep the focus on credibility and the aim of the study throughout the interview sessions. Other components to achieve trustworthiness in qualitative studies are transferability, dependability and confirmability. The parents in the study are described in order to achieve transferability to other studies in the research subject field. Quotes from different parents are represented in the study to show dependability. It was a challenge for the author of this study to enable confirmability, meaning to let the data speak for itself. The author strove to enable her own perspective from the data collection without giving meanings for subjects that were not included in the data. In order to achieve these perspectives, all of the study authors made their own interpretations of the data followed by discussions together that formed the data analysis.

The parents, when they were participating in the study, were no longer parents with children at the NICU, but observational studies looking at the actual care meetings between healthcare professionals and parents when language barriers exist would be of significance when further evaluation is done regarding the use of pictorial support.

## Conclusions

The present study sheds light on the challenges pertaining to communication within neonatal health care where language barriers exist. The parents described the communication as stressful and that misunderstandings were common. When a trustful relationship between the health care professionals and the parents were established, this was described to enable less feelings of isolation and anxiety and made the parents feel calmer. When pictorial support was used this increased understanding and decreased risks of misunderstanding. This study is the first to evaluate parents’ perspective from pictorial communication support distributed within the project KomHIT Flykting.
